# The flexible structure of the K24S28 region of Leucine-Rich Amelogenin Protein (LRAP) bound to apatites as a function of surface type, calcium, mutation, and ionic strength

**DOI:** 10.3389/fphys.2014.00254

**Published:** 2014-07-11

**Authors:** Jun-xia Lu, Sarah D. Burton, Yimin S. Xu, Garry W. Buchko, Wendy J. Shaw

**Affiliations:** Fundamental and Computational Sciences Directorate, Pacific Northwest National LaboratoryRichland, WA, USA

**Keywords:** amelogenin, LRAP, secondary structure, biomineralization protein, solid state NMR

## Abstract

Leucine-Rich Amelogenin Protein (LRAP) is a member of the amelogenin family of biomineralization proteins, proteins which play a critical role in enamel formation. Recent studies have revealed the structure and orientation of the N- and C-terminus of LRAP bound to hydroxyapatite (HAP), a surface used as an analog of enamel. The structure of one region, K24 to S28, was found to be sensitive to phosphorylation of S16, the only naturally observed site of serine phosphorylation in LRAP, suggesting that K24S28 may sit at a key region of structural flexibility and play a role in the protein's function. In this work, we investigated the sensitivity of the structure and orientation of this region when bound to HAP as a function of several factors which may vary during enamel formation to influence structure: the ionic strength (0.05, 0.15, 0.2 M), the calcium concentration (0.07 and 0.4 mM), and the surface to which it is binding [HAP and carbonated apatite (CAP), a more direct mimic of enamel]. A naturally occurring mutation found in amelogenin (T21I) was also investigated. The structure in the K24S28 region of the protein was found to be sensitive to these conditions, with the CAP surface and excess Ca^2+^ (8:1 [Ca^2+^]:[LRAP-K24S28(+P)]) resulting in a tighter helix, while low ionic strength relaxed the helical structure. Higher ionic strength and the point mutation did not result in any structural change in this region. The distance of the backbone of K24 from the surface was most sensitive to excess Ca^2+^ and in the T21I-mutation. Collectively, these data suggest that phosphorylated LRAP is able to accommodate structural changes while maintaining its interaction with the surface, and provides further evidence of the structural sensitivity of the K24S28 region, a sensitivity that may contribute to function in biomineralization.

## Introduction

Leucine-Rich Amelogenin Protein (LRAP) (Gibson et al., 1991) is a member of the amelogenin family of proteins, proteins which are known to be essential in the formation of enamel (Simmer and Fincham, 1995; Fincham et al., 1999; Margolis et al., 2006). The 59-residue protein is a splice-variant of full-length amelogenin and contains only the N- and the C-terminus (Gibson et al., 1991), regions associated with protein-protein and protein-HAP interactions in the full-length protein (Simmer and Fincham, 1995; Margolis et al., 2006). The role of LRAP in enamel formation is unclear, but it has been demonstrated to control both crystal organization *in vivo* (Gibson et al., 2008) and crystal width *in vitro* (Ravindranath et al., 2007), and may also serve a protein-regulating role (Stahl et al., 2013).

Structural studies of amelogenin have been challenged due to their self-assembly into quaternary structures containing 20–100 monomers called nanospheres (Fincham et al., 1994; Moradian-Oldak et al., 1994, 1995). These complexes, which form under many different conditions, are 500–1000 kDa in size and have never been crystallized, making structure determination using traditional solution-state NMR and X-ray diffraction (XRD) methods difficult. Solution studies at low pH, a condition that stabilizes the monomeric state, suggest amelogenin adopts few elements of canonical secondary structure, characteristic of intrinsically disordered proteins (Tompa, 2002; Uversky, 2002), making detailed structural analysis more complicated (Delak et al., 2009; Zhang et al., 2011; Buchko et al., 2013).

In recent years, solid state NMR (SSNMR) techniques have been extremely valuable for the quantitative structural characterization of biomineralization proteins in general (Drobny et al., 2002, 2003, 2006; Stayton et al., 2003; Goobes et al., 2007), and amelogenins in particular (Shaw et al., 2004, 2008; Shaw and Ferris, 2008; Masica et al., 2011; Lu et al., 2013a,b). Studying proteins bound to surfaces is inherently a solid state problem, and with the use of isotopic labels, SSNMR enables the site-specific investigation of structure, protein-surface interactions, and dynamics. Using such methods, LRAP has been extensively studied bound to hydroxyapatite (HAP), an analog for enamel which is described as carbonated apaptite (Simmer and Fincham, 1995; Fincham et al., 1999; Margolis et al., 2006), in order to identify structural features which may play important roles in binding to enamel (Shaw et al., 2004, 2008; Shaw and Ferris, 2008; Masica et al., 2011; Lu et al., 2013a,b). Many studies show that LRAP is a good model for the full-length protein (Moradian-Oldak et al., 1998b; Moradian-Oldak, 2001; Shaw et al., 2004, 2008; Le et al., 2006; Tarasevich et al., 2010) with the added advantage that its smaller size (59 residues) allows the incorporation of site-specific isotopic labels via solid phase peptide synthesis (Shaw et al., 2004, 2008; Shaw and Ferris, 2008; Masica et al., 2011; Lu et al., 2013b; Tarasevich et al., 2013). This has enabled quantitative structural studies of LRAP bound to HAP, providing much of the existing molecular level details of amelogenin's interactions with solid surfaces (Shaw et al., 2004, 2008; Shaw and Ferris, 2008; Masica et al., 2011; Lu et al., 2013b; Tarasevich et al., 2013).

Quantitative structural, dynamic, and orientation studies of LRAP bound to HAP include the following regions in the C- and the N-terminus: G8-Y12, L15-V19, V19-L23, K24-S28, L42-A46, A49-T53, and K54-V58 (Shaw et al., 2004, 2008; Shaw and Ferris, 2008; Masica et al., 2011; Lu et al., 2013b). In these pairs the first residue corresponds to a ^13^C-backbone carbonyl and the second a ^15^N-backbone amide. Collectively, the data on these LRAP samples bound to HAP represent the most comprehensive study of a biomineralization protein to date. In general, the C-terminus was found to be largely disordered and oriented close enough to the surface to influence mineral growth (5.8–8.0 Å), but at the same time is highly dynamic, suggesting that other regions of the protein may also be close enough to the HAP surface to stabilize interactions (Shaw et al., 2004, 2008; Shaw and Ferris, 2008). Investigations in the N-terminus revealed that this region contained much more helical content. The N-terminus was also similar in distance from or further from the HAP surface than the C-terminus depending on the phosphorylation state, 7.0–9.0 Å for LRAP(−P) and 5.3–7.0 Å for LRAP(+P), still close enough to the HAP surface in both cases to influence mineral growth. The variable distance suggests that in addition to interacting with HAP, the N-terminus may serve a second function such as facilitating protein-protein interactions (Masica et al., 2011). Neutron reflectivity studies bound to COOH-SAMs were also consistent with the N- and C-terminus of LRAP interacting with the surface (Tarasevich et al., 2013).

The only naturally observed site of serine phosphorylation in LRAP is at serine 16. To investigate the potential structural and functional implications of S16 phosphorylation, we recently completed SSNMR structural studies on the N-terminus of S16 phosphorylated LRAP and compared them to unphosphorylated LRAP. Using samples with pairs of site-specific labels (first residue a ^13^C-backbone carbonyl and the second a ^15^N-backbone amide), three regions covering the N-terminus, L15V19, V19L23, and K24S28, were studied bound to HAP as a function of solution condition. While some structural and dynamic variations in the L15–L23 region were noted, the most significant differences were observed in the K24S28 region (Lu et al., 2013b) a site 16 residues away from the phosphorylation. The sensitivity of the structure and dynamics of the K24S28 region to phosphorylation suggests that this may be a site with a potentially important role in biomineralization. Indeed, previous modeling studies suggest that K24 is a point at which the protein turns from the surface (Masica et al., 2011). In this work, we more fully investigate the structural and dynamic consequences on S16 phosphorylated LRAP by factors that may vary during enamel development: ionic strength (0.05, 0.15, and 0.2 M), Ca^2+^ concentration, and surface identity [HAP vs. carbonated apatite (CAP)]. A naturally occurring mutation found in full-length amelogenin (T21I) (Ravassipour et al., 2000) which results in malformed enamel was also investigated, due to its proximity to the K24S28 region. The implications of our results on the development of enamel will be considered.

## Experimental methods

### Materials

Labeled amino acids were purchased from Cambridge Isotopes (Andover, MA). Fmoc-protected labeled amino acids were prepared as previously described (Shaw et al., 2008) using standard procedures (Carpino and Han, 1972; Wiejak et al., 1999). The HAP (90 m^2^/g) used for binding was made and characterized according to literature preparation (Ebrahimpour et al., 1993) and stored as a slurry (28.9 mg/mL) to maintain the high surface area.

### Carbonated apaptite (CAP) synthesis and characterization

Solutions of calcium nitrate (1.18 g in 20 mL water) and sodium dihydrogen phosphate (0.41 g in 20 mL water) contained in dropping funnels were added simultaneously and dropwise at a rate of ca. 1 drop per second to a solution of sodium hydrogen carbonate (0.13 g in 20 mL) previously heated to 60 ± 5°C. The NaHCO_3_ solution was contained in a three-neck round bottom flask and was stirred magnetically. The pH was maintained at nine throughout the addition. When the addition was complete, the mixture was maintained at pH = 9 with stirring at 60 ± 5°C for 2 h. After the mixture cooled to room temperature it was vacuum filtered using a Büchner funnel and the residue was washed four times with distilled water. The product (0.91 g, 65% yield) was dried in a 120°C oven for 24 h. Powder XRD showed no evidence of calcium carbonate or other impurities. The XRD line pattern of the product was in good agreement with PDF-00-001-1008 HAP. The percent carbonate (5.2%) was determined by Galbraith Laboratories, Knoxville, TN by combustion in oxygen at 1000°C; the surface area was determined by the BET method to be 172 m^2^/g. All reagents were ACS reagent grade and distilled water was used throughout.

### Protein synthesis and purification

**Figure 1:**
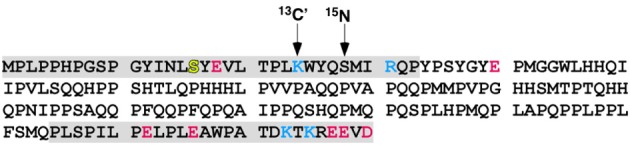
**Murine amelogenin amino acid sequence**. The primary amino acid sequence of murine amelogenin with the basic and acidic residues colored blue and red, respectively. The splice-variant LRAP is composed of the N-terminal 33 and C-terminal 26 residues, shaded gray. Both full-length amelogenin and LRAP are post-translationally modified by side chain phosphorylation of S16, shaded yellow. The isotopic labels at specific atomic locations and residues are shown with the arrows: K24 (^13^C′) and S28 (^15^N). In the T21I-mutant the threonine at position 21 is replaced with an isoleucine.

Site-specifically labeled LRAP-K24S28(+P) was prepared by solid phase peptide synthesis using Fmoc-chemistry by the Protein Chemistry Technology Center, University of Texas, (Dallas, TX). Isotopically labeled backbone carbonyl carbon at K24 (^13^C′) and amide nitrogen at S28 (^15^N) were introduced in the *i* and *i* + 4 positions, respectively (Figure 1). The T21I-mutant is LRAP-K24S28(+P) prepared with the substitution of threonine at position 21 with an isoleucine. The proteins were purified by reverse phase HPLC using: buffer A, 0.1% trifluoroacetic acid in water; buffer B, 0.1% trifluoroacetic acid in acetonitrile. Both proteins eluted at 54% B. Mass spectroscopy was used to characterize the purity and molecular weight of the proteins. After purification, proteins were lyophilized, and stored until ready for use.

### Protein solutions

LRAP-K24S28(+P) was bound to HAP under three different conditions and bound to CAP under the “standard condition,” while the T21I-mutant was bound to HAP under the “standard condition” (Table 1). The “standard condition” is defined as pH = 7.4, *IS* = 0.15 M, and 326 μg/mL (0.047 mM) LRAP. The solution for the standard conditions samples was prepared as follows: a buffer saturated with respect to HAP (Saturated Calcium Phosphate—SCP; the resulting solution has a Ca^2+^ concentration of 0.07 mM) was prepared by stirring excess HAP in a 0.15 M NaCl (pH 7.4) solution, maintaining pH. After 12–24 h, the undissolved HAP was filtered (0.22 μm, Isopore, Millipore). LRAP (16.8 mg) was then dissolved into 50 mL of SCP (326 μg/mL; 0.047 mM) and the pH adjusted to 7.4. For solutions with different ionic strengths, the same procedure was followed, but the NaCl concentration was adjusted to obtain the desired ionic strength (0.05 or 0.2 M). For the solution with eight-times excess calcium added relative to the LRAP concentration, the same procedure was followed, but CaCl_2_ was added to obtain 0.4 mM [Ca^2+^], providing a [Ca^2+^]:[LRAP-K24S28(+P)] of 8:1.

**Table 1:** **Sample conditions studied and their binding efficiency**.

**Condition**	**Binding (±4%)**
0.05 M ionic strength	68
0.2 M ionic strength	64
Bound to CAP	55
8-times excess Ca^2+^ (0.4 mM)	66
T21I-mutant	61
Standard conditions^*^	68^*^

* *Standard conditions previously reported: pH = 7.4, IS = 0.15 M, and 326 μg/mL LRAP (Lu et al., 2013b)*.

### Protein adsorption to hap or cap

The HAP or CAP was prepared for protein adsorption by washing 54.3 mg of HAP or CAP three times with 10 mL of SCP immediately before adding it to one of the above protein solutions. The mixture was stirred for 3 h at room temperature, centrifuged, and the LRAP-HAP or LRAP-CAP complex washed three times with 5 mL SCP to remove non-specifically bound protein. The amount of protein bound to HAP or CAP was determined by measuring the change in concentration before and after binding and for each wash using ultraviolet absorbance measurements (λ = 277).

The prepared sample was packed into the NMR rotor by first transferring it to a sealed 1 mL pipette tip. The tip was centrifuged for 10 min at 12,000 rpm to remove residual liquid, resulting in a tightly packed hydrated LRAP-HAP/CAP complex. The sealed end was then cut open and the pellet transferred to a 5 mm NMR rotor using a centrifuge. The rotor was placed in an NMR probe and spun at 6–7 kHz to remove the excess water, resulting in a >100% hydrated, surface bound sample. A thin layer of parafilm or a rubber disk was positioned before the rotor's end cap to keep the sample fully hydrated during NMR data collection.

### NMR experiments

All NMR experiments were conducted on a three-channel Chemagnetics Infinity spectrometer (Chemagnetics, Fort Collins, CO) with an Oxford 7.05 T [υ_0_(^1^H) = 300 MHz] wide-bore magnet, operating at resonance frequencies of υ_0_(^13^C) = 75.78 MHz and υ_0_(^15^N) = 30.54 MHz. Deconvolution of the 1D-spectra was performed with Mestranova (Mestrelab Research, Escondido, CA). For chemical shift measurements, a double resonance HX magic-angle spinning (MAS), variable temperature Chemagnetics probe was used. For REDOR measurements, a triple resonance HXY MAS, variable-temperature Chemagnetics probe was used. Temperatures in the rotor were calibrated using ^207^Pb(NO^3^)_2_ (Bielecki and Burum, 1995). Chemical shifts were referenced to the adamantane CH peak at 40.26 ppm (Wishart et al., 1995), which was referenced to 2,2-dimethyl-2-silapentane-5-sulfonate (DSS).

### Redor

XY8 phase cycling was applied on both observe (^13^C) and dephasing channels (^15^N or ^31^P) (Gullion and Schaefer, 1989, 1991). For all REDOR experiments, 180° pulses of 13.0–15.0 μs were used for both the observe and dephasing nuclei with a 65 kHz TPPM decoupling field (Bennett et al., 1995) employed during the recoupling and acquisition periods. Both ^13^C{^15^N} and ^13^C{^31^P} REDOR data were obtained for each sample preparation condition (Table 2). Data were acquired at −80°C to eliminate contributions due to motion and at a spinning speed of 4 kHz. For ^13^C{^15^N} REDOR, dephasing points were collected at 8, 24, 40, 56, 72, 88, and 104 rotor periods. Between 4096 and 8192 scans were taken for 8, 24, and 40 rotor periods, 8192–10240 scans for 56, 72, and 88 rotor periods, and 16384–20480 scans for 104 rotor periods. For ^13^C{^31^P} REDOR, dephasing points were collected at 8, 24, 40, 56, 72, 88, 104 rotor periods with 4096 scans taken for 8, 24, 40, and 56 rotor periods, and 8192–16384 scans for 72, 88, and 104 rotor periods. All data were collected with a 1 s pulse delay. Each point in the dephasing curve represents the average of at least five repetitions. The REDOR dephasing curves were corrected for the natural abundance background (59 backbone carbonyls and 10 side chain carbonyls, or 41% of the total signal) and were fit by simulations generated using SIMPSON (Bak et al., 2000). The best fit to the closest distance was determined utilizing a chi-squared analysis to provide a quantitative comparison of any given data set to the calculated dephasing curves.

**Table 2:** **^13^C′ chemical shifts for K24 in LRAP-K24S28(+P)**.

**LRAP-K24S28(+P) binding condition**	**Chemical shift (ppm) Downfield resonance (α-helix)**	**Chemical shift (ppm) Upfield resonance (β-strand/coil/background)**
Standard conditions	178.4 ± 0.5	174.8 ± 0.5
*IS* = 0.05 M	178.9 ± 0.5	175.1 ± 0.5
*IS* = 0.2 M	178.8 ± 0.5	175.1 ± 0.5
8:1 Ca^2+^:[LRAP]	178.9 ± 0.5	175.3 ± 0.5
Bound to CAP	178.7 ± 0.5	175.0 ± 0.5
T21I-Mutant	178.6 ± 0.5	174.8 ± 0.5

## Results

### Binding efficiency

As shown in Table 1, LRAP-K24S28(+P) bound to HAP with similar efficiency under the four conditions varied, where efficiency is the percentage of LRAP bound with respect to the initial amount available. The percent error is estimated based on the binding efficiency of multiple (>15) LRAP(+P) binding results. Slightly lower binding was observed for the high ionic strength condition and the T21I-mutant. Relative to HAP, LRAP-K24S28(+P) bound less efficiently to CAP.

### Chemical shift studies

**Figure 2:**
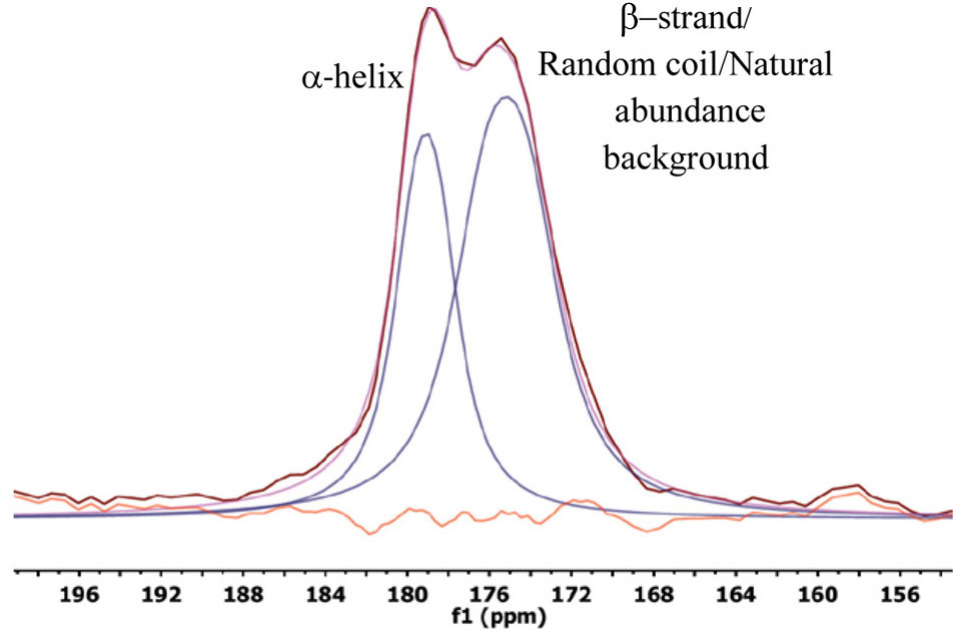
**One-dimensional ^13^C spectrum**. Representative spectrum of LRAP-K24S28(+P) bound to HAP, in this case at an ionic strength of 0.05 M. The deconvolution is also shown. The data is shown in pink, the combined fit in maroon, with individual fits in blue. The difference of the fit spectrum and the actual spectrum is shown in orange.

As illustrated in Figure 2 and Table 2, two distinct resonances are observed for the K24 carbonyl resonance in each sample that correspond to two different conformations. As previously described (Lu et al., 2013b), the downfield resonance is most consistent with a helical component of K24, while the upfield resonance is a combination of a β-strand component of K24, but also has contributions from the natural abundance (unlabeled) backbone and side chain carbonyls. A random coil component, if present, would be between the two resonances, but due to spectral overlap, we did not try to fit this separately. The chemical shifts of the helical carbonyl (Table 2) ranged from 178.4 to 178.9 ppm, while the upfield carbonyl ranged from 174.8 to 175.3 ppm, in both cases invariant within our experimental error to the varying binding conditions.

### Structure as a function of binding condition

**Figure 3:**
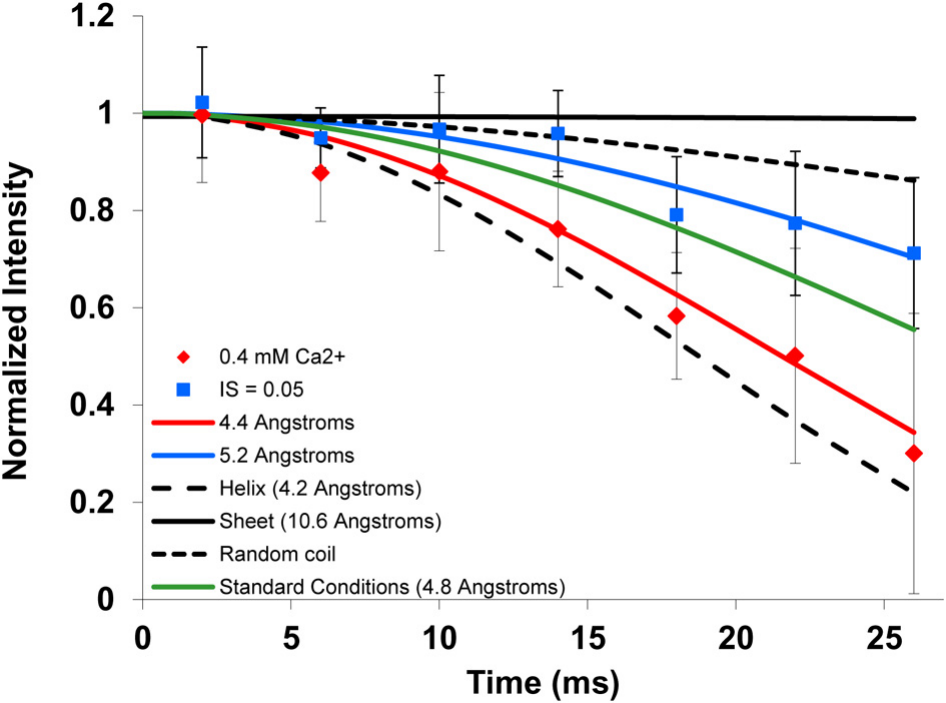
**Representative ^13^C-^15^N REDOR data and fits**. The ^13^C-^15^N REDOR data shows that LRAP-K24S28(+P) bound to HAP in the presence of excess Ca^2+^ (red diamonds) had the shortest C-N distance (4.4 Å, nearly helical), while binding at an ionic strength of 0.05 M (blue squares) resulted in a longer C-N distance (5.2 Å) consistent with a looser helix or less helical content at low ionic strength. The simulated dephasing curves for an α-helix (4.2 Å, dashed), β-strand (10.6 Å, solid), random coil (long dash), and the standard conditions (green line) are shown for comparison.

To gain further insight into the structural motif at K24 to S28, the distance between the ^13^C backbone carbonyl of K24 and the ^15^N amide of S28 was measured for each of the complexes using REDOR (Figure 3). The distances obtained from the resulting dephasing curves are well-established for regular α-helices (4.2 Å) and β-strands (10.6 Å). The dephasing curve for a random coil structure is obtained by averaging all of the dephasing curves between 4.2 Å and 10.6 Å, at 0.1 Å intervals, yielding an average distance of 5.8 Å. When bound to HAP under standard conditions, LRAP-K24S28(+P) had a significant helical content based on the ^13^C-^15^N distance of 4.8 Å. As seen in Table 3, this distance is unchanged when bound at an ionic strength of 0.2 M, or for the T21I-mutant bound under standard conditions, indicating a similar content of α-helix for these conditions. When bound at an ionic strength of 0.05 M, the distance lengthens to 5.2 Å (Figure 3), indicating a shift to more random coil structures, and suggesting that salt concentration influences structure. Under conditions where the protein is bound to CAP or bound in the presence of 0.4 mM Ca^2+^, the distance decreases to 4.4 Å (Figure 3), a value approaching a canonical α-helix, suggesting that calcium may stabilize helix formation in this region of LRAP.

**Table 3:** **REDOR determined distances for LRAP-K24S28(+P)**.

**LRAP-K24S28(+P) binding condition**	**^13^C(*i*)-^15^N(*i* + 4) distance**	**^13^C-^31P^ distance**
Standard condition	4.8 ± 0.4 Å	7.5 ± 0.5 Å
*IS* = 0.05 M	5.2 ± 0.5 Å	8.0 ± 0.5 Å
*IS* = 0.2 M	4.9 ± 0.4 Å	8.0 ± 0.5 Å
8-times calcium (0.4 mM)	4.4 ± 0.3 Å	8.5 ± 0.5 Å
Bound to CAP	4.4 ± 0.3 Å	7.5 ± 0.5 Å
T21I-Mutant	4.8 ± 0.4 Å	8.5 ± 0.5 Å

The dephasing curves can also result from a combination of multiple distinct structures. In the case of dephasing curves consistent with α-helices [e.g., LRAP-K24S28(+P) bound to CAP or bound to HAP in the presence of 0.4 mM calcium], the contribution from structures other than α-helix would be low. However, for dephasing curves with longer distances which are indicative of structures deviating from a helical structure, contributions from random coil or β-strand structures can be significant. For example, at an ionic strength of 0.05 M, the dephasing curve fits to a single distance of 5.2 Å, but could be fit equally well by some contribution of α-helix, random coil, and/or β-strand. To provide a starting point, deconvolution and integration of the 1D-spectra (Figure 2) suggests that ~60% of this region is in an α-helical structure. This number is based on integrations of 37% α-helix (downfield signal) and 23% other (upfield signal) with the later signal corrected by removing the 40% intensity contribution due to the natural abundance ^13^C′ background. This analysis is not quantitative due to potential errors in deconvoluting as well as other possible contributions to the intensity of the resonance attributed to the α-helical component. Fixing the α-helical contribution to 60% resulted in a curve that dephased much faster than the data, regardless of whether the remaining 40% was random coil or β-strand. However, a combination of 40% α-helix, 60% β-strand fits the data well, as does 30% α-helix, 70% random coil. These two options cannot be distinguished, nor can they be distinguished from a combination of all three structures (40% α-helix, 10% random coil, and 50% β-strand) that fit the data. What is clear for the combined chemical shift and REDOR data is that the K24S28 region has a smaller population of α-helical character as a function of low ionic strength.

### Distance from the surface as a function of binding condition

The ^31^P nucleus on the HAP surface provides another handle with which to characterize the HAP-LRAP interface. This is because the distance from the backbone ^13^C carbonyl to surface ^31^P can be measured, providing an indication of the orientation of the protein relative to the surface. For instance, if the K24 carbonyl is oriented away from the surface, the ^13^C′-^31^P distance would be long (>12 Å), while a strong interaction in this region would result in a much shorter distance. The shortest distance from the backbone ^13^C′ observed for LRAP to date is 5.3 Å (Masica et al., 2011).

**Figure 4:**
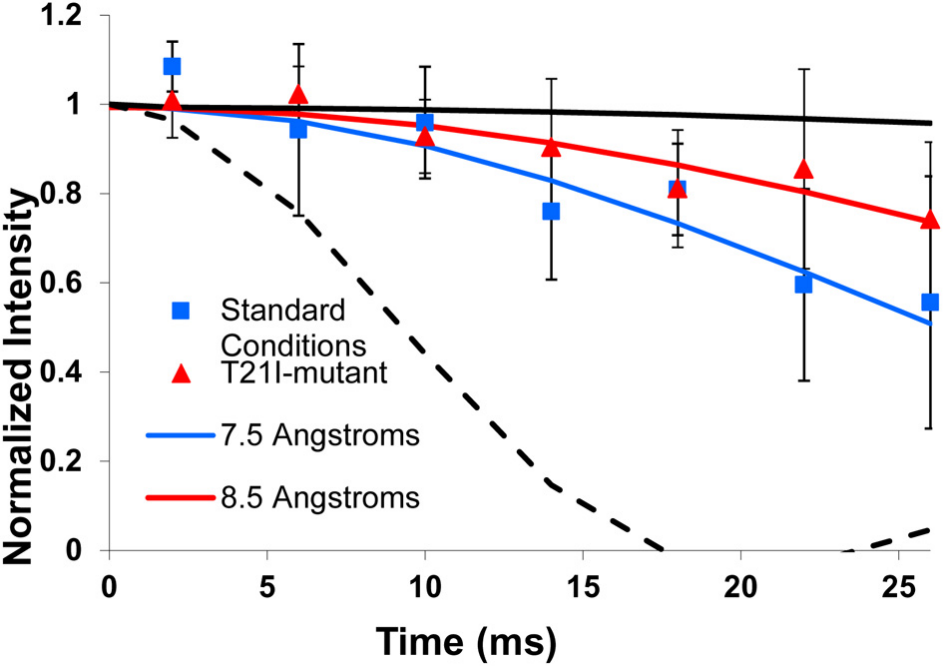
**Representative ^13^C-^31^P REDOR data and fits**. The ^13^C-^31^P REDOR data for LRAP-K24S28(+P) under the standard condition (blue squares) and for the T21I-mutant (red triangles) with fits for a distance of 7.5 Å (blue line) and 8.5 Å (red line). The T21I-mutant and the sample prepared in the presence of 0.4 mM Ca^2+^ (not illustrated) showed the largest ^13^C-^31^P distance difference relative to the standard condition (~1 Å). A distance too long to measure (12 Å, solid black line) and the shortest distance measured from a backbone ^13^C′ to HAP (5.3 Å, dashed line) are shown for reference.

Binding under standard conditions resulted in a ^13^C-^31^P distance of 7.5 ± 0.5 Å from the nearest ^31^P on the surface (Figure 4 and Table 3). Binding to CAP and to HAP at an ionic strength of 0.05 or 0.2 M did not result in any ^13^C-^31^P distance changes relative to the standard condition outside the error limits of the measurements. The largest ^13^C-^31^P distance changes relative to the standard condition were observed for LRAP-K24S28(+P) in the presence of 0.4 mM Ca^2+^ and for the T21I-mutant, which both increased to 8.5 ± 0.5 Å (Figure 4). Interestingly, of the latter two conditions, a change in structure was only observed in the presence of excess calcium.

## Discussion

The amelogenin proteins have been classified as intrinsically disordered (Delak et al., 2009), a characterization which implies structural flexibility may be necessary to achieve a particular function (Tompa, 2002; Uversky, 2002). Consistent with a predilection to structural flexibility, both amelogenin and LRAP are dynamic even when they are bound to HAP (Shaw and Ferris, 2008; Shaw et al., 2008; Masica et al., 2011; Lu et al., 2013a,b). Despite the inherent flexibility, specific structured regions have been identified for the surface immobilized protein based on SSNMR REDOR data (Masica et al., 2011; Lu et al., 2013a,b). The LRAP-K24S28(+P)/(−P) has been identified to be an ideal helix when lyophilized from solution. Upon binding to HAP, LRAP(+P) remained helical, while LRAP(−P) became largely unstructured. The observation of structural lability as a function of phosphorylation suggests an important function for this region of the protein.

Protein structure in solution state NMR is often evaluated using ^13^C, ^15^N, and ^1^H chemical shifts. This is because NMR chemical shifts arise from the electron density around the nucleus and differs for each element of secondary structure (α-helix, β-strand, random coil). For the same reasons, chemical shifts can also provide qualitative insight into the structure of proteins immobilized onto surfaces in the solid state. In this case, the unique observation of two resonances for the carbonyl of K24 compared to one resonance for all of the other carbonyls studied for LRAP (Shaw et al., 2004, 2008; Shaw and Ferris, 2008; Masica et al., 2011; Lu et al., 2013a,b) suggests a unique and important structural feature for this region of the protein. We previously demonstrated that the downfield K24 carbonyl resonance is most consistent with an α-helical conformation, based on the downfield chemical shift expected for a helical carbonyl and the evaluation of REDOR data for this resonance alone (Lu et al., 2013b). That two K24 carbonyl resonances are observed under each of the preparation conditions tested (Table 2) suggests that there are common structural features, including a significant amount of helical structure at K24, regardless of the preparation.

In addition to secondary structure, for surface immobilized proteins, differences in chemical shift can also arise from how the protein is associated with the highly charged surface due to alterations of local electron density. Consequently, the chemical shift and intensity measurements for the two K24 carbonyl resonances are qualitative and the interpretations need to be treated with care. The difficulty in quantifying chemical shift information for surface immobilized proteins due to these two contributions emphasizes the need for a more quantitative technique. Dipolar recoupling techniques such as REDOR, which directly measure the distance between two nuclei, provide such quantitation.

The REDOR data under the binding conditions studied here provide further evidence for a structurally flexible region centered around the K24S28 region that may play a functional role. For example, the REDOR data is consistent with an ideal α-helix in this region in the presence of excess Ca^2+^ (8:1 [Ca^2+^]:[LRAP-K24S28(+P)]), suggesting a significant increase in helical content in this region relative to binding under the standard condition. Associated with this increase in helical character is a 1 Å increase in the K24 carbonyl distance to ^31^P on the surface. Since soluble free calcium is present *in vivo* in the enamel fluid at similar concentrations to those used in our studies (Aoba and Moreno, 1987), this is a critical observation and suggests the importance of calcium in dictating structure. Previously, small angle X-ray scattering was used to investigate the overall shape of LRAP(+P) and LRAP(−P) in solution as a function of Ca^2+^ concentration. Under Ca^2+^:[LRAP] ratios similar to ours, significant structural changes were also observed with LRAP(+P) transitioning from an extended to a more globular structure as a function of increasing Ca^2+^ (Le Norcy et al., 2011a). While the X-ray scattering and sedimentation experiments do not have the resolution to identify molecular level structural changes, the microscopic changes observed here using SSNMR are in agreement with the macroscopic changes observed using sedimentation velocity and X-ray scattering and may be providing the first insight into the reasons behind the macroscopic changes. Further comparisons suggest a competition between solubilized calcium and calcium in the HAP lattice that impact structure: LRAP-K24S28(+P) had a distance consistent with an ideal helix (measured distance was 4.2 ± 0.3 Å) when lyophilized from solution with 0.007 mM Ca^2+^ in solution. When LRAP-K24S28(+P) was bound to HAP, a loss of helical structure was observed unless excess solution calcium (0.4 mM Ca^2+^) was present. These observations suggest that the structural changes resulting from binding solution calcium modify the protein:surface interface. Given the observed structural change, a reasonable expectation would be different surface coverages in the presence or absence of Ca^2+^, however, similar surface coverage by the protein was obtained. Since the observed structural change does not control the amount of protein binding, the structural change resulting from excess solution Ca^2+^ must serve another function, such as activating protein binding or mineral growth. Indeed, protein-protein interactions were observed to increase for LRAP(+P) in solution in the presence of excess solution Ca^2+^ (Le Norcy et al., 2011a), and these interactions may play an important role for the surface immobilized protein.

Although the majority of studies of amelogenin interactions with crystal surfaces have involved HAP (Moradian-Oldak et al., 1998a,b, 2002; Habelitz et al., 2006; Wang et al., 2007; Shaw and Ferris, 2008; Shaw et al., 2008; Kwak et al., 2009, 2011; Tarasevich et al., 2009; Le Norcy et al., 2011a,b; Masica et al., 2011; Uskokovic et al., 2011; Lu et al., 2013a,b), CAP is a more direct analog of enamel (Simmer and Fincham, 1995; Fincham et al., 1999; Margolis et al., 2006). The structure at LRAP-K24S28(+P) is also consistent with an ideal α-helix when bound to CAP (4.4 ± 0.3 Å), in contrast to HAP where the structure is shifted toward random coil (4.8 ± 0.4 Å). This is some of the first data suggesting that carbonation influences the structure of amelogenins, and therefore, could influence the resulting mineral growth.

The T21I-mutant is one of many naturally occurring, single-site mutations found in full-length amelogenin that results in enamel hypoplasia, with enamel features including smooth pits, variable thickness, mottled, and brown color (Ravassipour et al., 2000). That this significant loss of functional enamel results from a single-site mutation strongly suggests a change in structure, and changes in tertiary structure (protein-protein interactions) have been observed (Moradian-Oldak et al., 2000; Paine et al., 2002; Buchko et al., 2013). However, no structural change for the T21I-mutant was observed in LRAP-K24S28(+P), despite the proximity of the mutated residue to the K24S28 region. The mutation did result in the region being further from the surface and a simple interpretation for this observation is that the threonine in the native protein assists in holding the protein closer to the surface and replacement with a hydrophobic isoleucine destabilized this interaction. The distance from the surface for the T21I-mutation determined under standard conditions is similar to that found when excess Ca^2+^ was present, though it is unclear at this time if there is a mechanistic correlation between these two observations.

The functional importance of the structural flexibility in the K24S28 region is not clear and further crystal growth and structural studies will be necessary to tease out the mechanistic details. An equally important observation is that the structure doesn't completely switch conformations, i.e., under all conditions studied, there is still some helical character. This could merely be a result of the high entropic costs associated with switching structures when the protein is immobilized. Given the uniqueness of this structured region in LRAP, a more likely explanation is that it is a critical part of the functional design, possibly serving a regulatory role in enamel development. Only the continued *in vitro* investigation of the structural and functional properties of the amelogenin proteins will allow us to fully explore these mechanisms.

## Summary

The LRAP(+P):HAP interaction of the structurally flexible K24S28 region was evaluated as a function of ionic strength, calcium concentration, surface type, and protein mutation. Modest differences were observed in both structure and distance from the surface for this region of the protein. The most significant difference, relative to the standard condition, was observed with LRAP bound to HAP in the presence of excess Ca^2+^, where the protein in this region transitioned from a looser α-helix to a nearly ideal α-helical structure and moved further from the surface. The dependence of LRAP structure (secondary, tertiary) on calcium concentration is mounting, and these studies have provided some of the first insights at the molecular level. Using a CAP surface also resulted in a more ideal helical structure, while the T21I-mutation resulted in moving the K24S28 region further from the surface. Collectively, these data provide continued evidence of the structural flexibility of the K24S28 region in amelogenins and further implicated it as a functionally important region.

### Conflict of interest statement

The authors declare that the research was conducted in the absence of any commercial or financial relationships that could be construed as a potential conflict of interest.
